# Surface Plasmon Resonance or Biocompatibility—Key Properties for Determining the Applicability of Noble Metal Nanoparticles

**DOI:** 10.3390/ma10070836

**Published:** 2017-07-21

**Authors:** Ana Maria Craciun, Monica Focsan, Klara Magyari, Adriana Vulpoi, Zsolt Pap

**Affiliations:** 1 Nanobiophotonics and Laser Microspectroscopy Center, Interdisciplinary Research Institute on Bio-Nano-Sciences, Babeș-Bolyai University, 400271 Cluj-Napoca, Romania; ana.gabudean@ubbcluj.ro (A.M.C.); monica.iosin@phys.ubbcluj.ro (M.F.); 2 Nanostructured Materials and Bio-Nano-Interfaces Center, Interdisciplinary Research Institute on Bio-Nano-Sciences, Babeș-Bolyai University, 400271 Cluj-Napoca, Romania; adriana.vulpoi@phys.ubbcluj.ro; 3 Institute of Environmental Science and Technology, University of Szeged, 6720 Szeged, Hungary

**Keywords:** bioactive glasses, noble metal nanoparticles, surface plasmon resonance, plasmonic biosensors, diagnostics, photocatalysis, composite photocatalysts, photoactivity

## Abstract

Metal and in particular noble metal nanoparticles represent a very special class of materials which can be applied as prepared or as composite materials. In most of the cases, two main properties are exploited in a vast number of publications: biocompatibility and surface plasmon resonance (SPR). For instance, these two important properties are exploitable in plasmonic diagnostics, bioactive glasses/glass ceramics and catalysis. The most frequently applied noble metal nanoparticle that is universally applicable in all the previously mentioned research areas is gold, although in the case of bioactive glasses/glass ceramics, silver and copper nanoparticles are more frequently applied. The composite partners/supports/matrix/scaffolds for these nanoparticles can vary depending on the chosen application (biopolymers, semiconductor-based composites: TiO_2_, WO_3_, Bi_2_WO_6_, biomaterials: SiO_2_ or P_2_O_5_-based glasses and glass ceramics, polymers: polyvinyl alcohol (PVA), Gelatin, polyethylene glycol (PEG), polylactic acid (PLA), etc.). The scientific works on these materials’ applicability and the development of new approaches will be targeted in the present review, focusing in several cases on the functioning mechanism and on the role of the noble metal.

## 1. Introduction

Noble-metal nanoparticles present an extremely widened application spectrum, which cannot be expressed efficiently in numbers. Therefore, to gather relevant knowledge and to present information to the large scientific audience, specific in-depth, or applicability spectrum-based review papers are needed. Consequently, the following main interest areas were considered and covered in this work:

(1) development of innovative design of plasmonic biosensors with highly sensitive, selective, and reliable biomarker detection abilities to enable early diagnosis and improved disease treatment.
(2) tissue engineering and drug delivery systems based on noble metal nanoparticles in bioactive glasses and glass ceramics: the specific biological effect of these materials such as silver nanoparticles are described by the antibacterial activity and healing enhancement effect of nano-silver, copper nanoparticles also demonstrated the size-dependent antibacterial activity with low toxicity, while bioactive glasses with gold nanoparticles have good proliferation rate of keratinocytes cells.
(3) plasmonic effect-based photocatalysis: the surface plasmon resonance (SPR) of some noble metals can be exploited to activate photocatalytic materials, by injecting hot electrons in to the conduction band of semiconductors making possible visible- and near infrared light driven degradation of organic pollutants.

As it was described above, the chosen application areas are well separated (however, cross-linked by the two mentioned properties: biocompatibility and SPR). Therefore, it is very interesting to see how the mentioned properties were exploited, investigated and described in different research areas. Furthermore, the present review paper will focus mostly on gold and silver in to emphasize the weight of a noble metal in the specific research area.

## 2. Optical Plasmonic Biosensors for Smart Disease Diagnostics

Timely detection of specific disease biomarkers is one of the top priorities in order to enable early diagnosis and improve diseases treatment [1]. Presently, the majority of current clinical diagnostic methods rely on the identification and quantification of disease biomarkers [2]. A biomarker is “*a biological molecule found in blood, other body fluids, or tissue that is a sign of a normal or abnormal progress or of a condition or disease*”, as the National Cancer Institute defines [3]. Most of currently employed diagnostic procedures are long, complex and invasive processes which imply sophisticated assays, including multi-step protocols and difficult fluid handling. A nowadays research priority in the field of biomedical diagnostic is focused on the development of point-of-care (POC) procedures to allow rapid determination of relevant analytes directly in the doctor’s office or the emergency ward, where regular assays for protein analytes, such as enzyme-linked immunosorbent assay (ELISA), radio/electrophoretic and mass spectrometry (MS)-based proteomics cannot be performed. Concretely, many disease biomarkers are proteins and their presence in biological fluids is considered an indicator of the presence of some diseases such as diabetes, cancers, etc. One of the most common methods to get information about proteins in the body is the fluorescence spectroscopy, which usually requires labeling of proteins with fluorescent substances [4]. The main drawbacks of this approach are: (a) the complexity of the chemical process of labeling which also induced alteration of the proteins or the biological processes under investigation and (b) the rapid process of photobleaching (photochemical destruction of the fluorophore) underwent by the fluorescent label, which limits the examination time. Another current method used for detection of protein biomarkers in diagnostic is the ELISA assay, which however requires labeling of the proteins, complex optical equipment, and significant technical expertise, making this technique expensive and time consuming. Despite the detection limits approaching 1 pg∙mL^−1^ for some biomarkers [5], this technique is difficult to employ for POC use. Considering these points, an urgent and critical step toward the implementation of smart and early medical diagnostic tools is represented by the development of inexpensive and user-friendly diagnostic nanoplatforms for the detection of various biomarkers with a very high sensitivity, selectivity and reliability. Trying to respond of this desideratum, a myriad of well-designed biosensors for optical detection of specific cancer biomarkers has appeared in the literature during the last decades. Additionally, the researchers demonstrated that by the integration of these plasmonic biosensors into microfluidic circuit it is possible to obtain a single biochip (so-called “lab-on-chip”) able to detect very low concentrations of biomarkers in a minimum volume of sample collected from simulated or biological fluids, developing thus a portable and miniaturized POC devices for mass utility [6,7,8,9]. In particular, the development of such integrated plasmonic biosensor in microfluidic device has the real potential to push forward the smart medical diagnostic by: (a) reducing the detection limits of relevant biomarkers; (b) allowing an early diagnosis of some diseases; (c) tracking the presence of proteins in real time in body fluids; (d) introducing less- or non-invasive procedures in medical diagnostic; and (e) allowing fast and ultrasensitive clinical analysis. Furthermore, microfluidic devices are very appealing systems for sensitive biomarkers detection due to the decreased risks of contaminating the biological samples, the extremely small sample volumes (10^−9^–10^−8^ L) required and—more exciting—the possibility for developing high-throughput, real-time, parallel, and multiplexed analyses [10], all these aspects having a major impact on overall nanosensor performance and capture kinetics.

In recent years, significant attention was paid to designing innovative optical biosensors based on plasmonic transducers with greater efficiency to translate the system into clinical use, where the main limitation is its modest detection sensitivity. For example, ELISA is the current gold standard for clinical prostate-specific antigen (PSA) biomarker detection, with a limit of detection of 0.1 ng∙mL^−1^ [11]. However, this detection limit is higher than the concentrations of cancer biomarkers in clinical serum samples, which are sometimes useless when an early stage detection of the disease is desired. Thanks to the development of nanotechnology, gold (Au) nanostructures have drawn a considerable interest as plasmonic transducers, because of their unique physical and optical properties related to their size, shape and dielectric microenvironment [12], which make them excellent scaffolds for the development of biosensors for a variety of target biomarkers. In fact, their optical properties are governed by Localized Surface Plasmon Resonance (LSPR), which is the result of the collective oscillations of the metal conduction electrons after being exposed to a light beam [13]. The spectral sensitivity of LSPR to the dielectric properties of environment has recently started to be investigated in plasmonic-based immunoassays, thus enabling the sensitive and specific label-free ultradetection of target biomarkers of interest. In the case of disease diagnostics, the LSPR detection takes place when a measurable wavelength red-shift of the plasmonic band caused by changes in the local refractive index around the metallic surface is detected [14]. More importantly, excepting all the advantages above-mentioned, Au nanostructures-based biosensors are highly favored for development by researchers because of their surface versatility which provides many possibilities for functionalization. Additionally, the capability to finely tune their surface properties, size, shape as well as their aggregation state (in case of colloidal Au nanoparticles (AuNPs)), makes Au nanostructures ideal biosensor platforms, that can be successfully employed for specific diagnostic applications. Therefore, in the following section we will focus on providing an overview on the recent progress in employing different types of Au-based biosensors—both solid and in colloidal suspension—with the aim to improve and enlarge their applicability in smart disease diagnostics.

### 2.1. Solid Plasmonic Substrate-Based Immunoassays 

Various Au-based biosensors—as solid plasmonic platforms—have been designed in the literature using different innovative fabrication strategies and tested in terms of sensitivity and limit of the detection (LOD) for detection of disease-related specific biomarkers. For example, Troung et al. [15] successfully fabricated an individual Au nanorods-based immunosensor immobilized on glass substrate for the LSPR detection of PSA-antichymotrypsin (PSA–ACT complex) at concentrations as low as 111 attomolar. The improvement of the detection sensitivity is in fact the key of early diagnostics and better treatment of specific diseases. However, to reach a higher sensitivity than with conventional ELISA, researchers have also focused their attention on developing new fluorescence-based immunoassays strategies that have the potential to revolutionize smart clinical detection. For example, Liu el al. [16] developed an activatable and ultrasensitive probe based on Rhodamine B isothiocyanate (RBITC) loaded onto AuNPs for detection of PSA in patient serum samples with high sensitivity. The new as-formed RBITC-AuNP conjugates induced a complete fluorescence quenching of RBITC dye molecules through the Nanometal Surface Energy Transfer (NSET) mechanism. Then, this positively charged complex was linked to the negatively charged Ab2 antibodies via electrostatic interaction, retaining in this way their biological activity towards the target PSA antigen. In order to form a sandwich structure, the Ab2- RBITC-AuNP complex was pulled down onto the surface and after subsequent addition of cysteamine, the loaded dyes were immediately released from the AuNPs surface, and consequently the fluorescence intensity of RBITC was enhanced. The recovered fluorescence intensity was associated with the concentration of PSA spiked in serum samples, obtaining a detection limit down to 0.032 pg∙mL^−1^.

**Figure 1:**
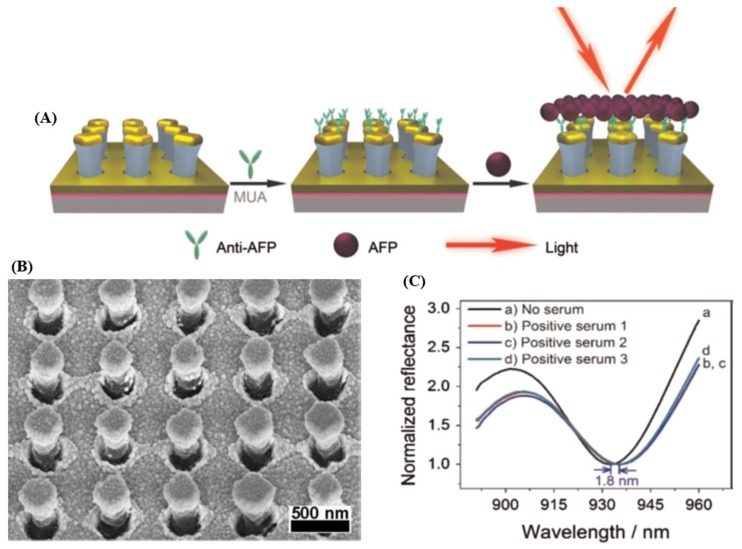
(**A**) Schematic illustration presenting the covalent functionalization of the fabricated plasmonic biosensor with anti-alpha-fetoprotein (AFP) for label-free and one-step localized surface plasmon resonance (LSPR) detection of AFP; (**B**) Representative SEM image of the fabricated plasmonic substrate; (**C**) Normalized reflectance spectra of the plasmonic biosensor before (a) and after (b–d) exposure to human serum sample three times. (Reproduced with permission from Reference [17] published by Elsevier).

Recently, Li et al. [17] reported on the fabrication of Au mushroom-based array nanosensors using the interference lithography (IL) for the label-free, one-step specific detection of alpha-fetoprotein (AFP) (see Figure 1A), an important biomarker specific to hepatocellular carcinoma in clinical patient serum. Specifically, the morphology of the as-fabricated plasmonic substrate via IL and Au deposition is presented in Figure 1B. The LOD determined by AFP antibody detection in buffer solution (here in 24 ng∙mL^−1^) was found to be below the critical concentration in normal plasma, which was established to be ≈25 ng∙mL^−1^. Furthermore, in order to demonstrate the feasibility of the fabricated biosensor, the AFP detection was evaluated using real serum samples collected from different patients suffering from liver cancer, the obtained LSPR results being consistent with the clinical ones obtained by electrochemiluminescence immunoassay. Concretely, the recorded reflectance spectrum of the plasmonic substrate shifts ≈1.8 nm for a positive clinical sample, like 90 ng∙mL^−1^ according to the calibration curve (Figure 1C).

More significantly, for improved healthcare system where multiple biomarkers should be simultaneously detected, it is highly desirable to develop innovative and convenient nanosensors, which are amenable for multiplexed detection. With this aim, Sim et al. [18] have recently designed novel nanoplasmonic chip-based biosensors for the label-free specific detection and quantification of three different targeted cancer biomarkers (i.e., AFP, carcinoembryonic antigen (CEA) and PSA from patient-mimicked serum samples), chosen herein as a “proof-of-concept”. The proposed multiplex plasmonic biosensor was fabricated by immobilizing in a selective manner the targeted immune-AuNPs on a hydrophilic-hydrophobic patterned microscope slide. The LSPR response of the immune-AuNPs is shifted to longer wavelengths as a function of the concentration of cancer biomarkers, indicating the success formation of biomarker@AuNPs complex. As a result, the LODs of the developed sensing platform were determined to be 91 fM, 94 fM and 10 fM, values suitable for direct implementation of the platform in practical clinical detection. The advantage of this platform is that it can be easily extended to detect other relevant cancer biomarkers for the clinical diagnostics field.

In the same period, Lee et al. have investigated the possibility to develop a label-free immunoassay by fabricating a fiber-optic LSPR nanosensor for the real-time detection of PSA with high resolution and sensitivity [19]. In this case, the nanosensor was fabricated by using spherical AuNPs immobilized on the end-face of the optical fiber via the self-assembled monolayer approach. The authors have concluded that the proposed fiber-optic LSPR nanosensor can be further applied for clinical testing, being able to detect a PSA concentration as low as 1 pg∙mL^−1^. Recently, an important progress towards the development of the portable and miniaturized label-free optical-fiber LSPR technology has been realized by achieving the lowest LOD at 3 fM of free PSA in PBS buffer solution [20]. For PSA detection, a very stable and robust fiber biosensor has been fabricated by an inexpensive lift-off process adapted to design Au nanodisk arrays at the fiber end facet. However, just keeping these examples in mind, it is important to note that—in general—the development of fiber-optic based on LSPR technology is important for POC applications due to its relatively simple optical set up with no electromagnetic interference, low clinical sample volume used, low-cost, as well as—very importantly for a real-time and label-free clinical sensing—portability and miniaturization [21].

More recently, an innovative analytical label-free strategy has been proposed by Lechuga’s group [22] for the rapid detection and real-time quantification of tumor-associated autoantibodies (TAA) directly in clinical blood serum and plasma for non-invasive diagnosis of colorectal cancer (CRC) at early stages. Specifically, the developed refractometric nanobiosensor is based on the LSPR response of Au nanodisks fabricated by hole-mask colloidal lithography technique, which allowed us to obtain uniform and reproducible plasmonic biosensors. After rigorous optimization of the biofunctionalization protocol based on the formation of an alkanethiol self-assembled monolayer onto the plasmonic nanodisks, an LOD of 1 nM has been reached (i.e., 150–160 ng∙mL^−1^). The excellent sensitivity and reproducibility of the designed nanobiosensor together with its simplicity of detection (without specific labels or sample pretreatments) provide a real non-invasive POC alternative that could be implemented for reliable CRC diagnostics.

Going further, flexible platform-based technologies, in particular paper-based nanosensors, have recently created an exciting avenue in the field of portable POC diagnostics [23]. Paper-based nanosensors represent an attractive, innovative, and inexpensive plasmonic detection nanoplatforms owing to their multiple advantages such as low-cost and easy fabrication, ease of use, high specific surface area, flexibility, or excellent wicking ability due to capillary forces. Excellent articles have been focused on the immobilization of plasmonic NPs by immersion approach onto various types of paper, like filter paper [24], photocopy paper [25], poly(L-lactic acid) nanofibrous paper [26] or cellulose paper [27].

Tian et al. [28] have developed for the first time an inexpensive, environmental-friendly, and highly sensitive plasmonic nanotransduction platform using a common laboratory filter paper (i.e., Whatman 1) for the rapid and label-free LSPR detection of aquaporin-1 (AQP1) protein in artificial urine sample, an important cancer biomarker for early detection of renal cancer carcinoma (RCC). The developed bioplasmonic paper-based platform consists in Au nanorods conjugated firstly with anti-AQP1 and then adsorbed on the filter paper. By monitoring the LSPR shift by increasing the concentration of AQP1, an LOD of 10 ng∙mL^−1^ was noted. This value is well-matched with the lower limit of the range of AQP1 in patients with kidney cancer. Due to its three-dimensional (3D) porous structure compared to glass solid substrate, this type of flexible paper-based LSPR substrate facilitates a better uptake and transport of targeted biomarkers, enabling a larger LSPR shift and consequently the possibility to achieve a lower LOD.

Tadepalli et al. [29] have demonstrated the selective and sensitive LSPR detection of the cardiac biomarker troponin I (cTnI), an important indicator for the detection of myocardial damage, using a bioplasmonic paper device, as a sensing plasmonic nanoplatform. Specifically, with the aim to directly translate the LSPR-based paper biosensor to inexpensive and rapid POC diagnostics, the authors functionalized the Au nanorods treated paper with peptide recognition elements with high affinity for cTnI biomarker. A major advantage of employing short peptides as recognition elements compared to larger antibodies was their enhanced chemical and environmental stability, making peptide-based LSPR sensors an excellent candidate for POC diagnostics, particularly for the resource-limited settings. Considering all these advantages, troponin was detected in this way directly in complex physiological fluids, achieving an LOD in human plasma of 353 pg∙mL^−1^. The ultrasensitive detection of troponin has also been achieved from artificial eccrine sweat at physiologically relevant concentrations [30].

The major advantage of Surface enhanced Raman spectroscopy (SERS) technique—as a powerful fingerprinting tool—relies on its ability to provide rich and complex spectroscopic information for the ultrasensitive detection of biomarkers, as well as their identification without labeling the targeted analytes. Additionally, compared to conventional detection techniques like MS, which offers high sensitivity but implies purification of the protein sample before analysis [31], or radioactive immunoassays which are less time consuming, SERS can be used as successful POC tool due to its multiplexing detection capability, single biomarker sensitivity and ease-of-use without complicated sample preparation. In general, to perform a reliable, fast diagnosis and imaging of diseases, e.g., cancer (margins) or infectious diseases, analytical methods allowing a specific and sensitive detection are required. SERS can be such a tool, since molecular specific Raman spectroscopy is combined with high sensitivity based on exceptional plasmonic properties of metallic nanostructures (i.e., field enhancement). Up to now, theoretical articles demonstrated that the maximum electromagnetic field enhancement in SERS occurs specifically in between NPs or in close proximity to nanometer-sized metallic nanostructures [32]. In fact, metallic nanostructures can be modified with a reporter molecule and specific recognition units creating SERS labels for the specific interaction of binding sites, producing an important enhancement (10^4^–10^8^) of the Raman spectrum [33].

In the work presented by Wu et al. [34], a novel sensitive SERS immuno-sensor has been constructed for protein biomarker detection, employing a periodic Au triangle nano-array platform coupled to Au nanostar@Raman-reporter@SiO_2_ sandwich NPs. Using this coupling strategy, a large number of “hot-spots” can be generated in between the as-created 3D space. This biosensor demonstrated the ability to measure the level of vascular endothelial growth factor (VEGF) in human blood plasma samples taken from the breast cancer patients.

**Figure 2:**
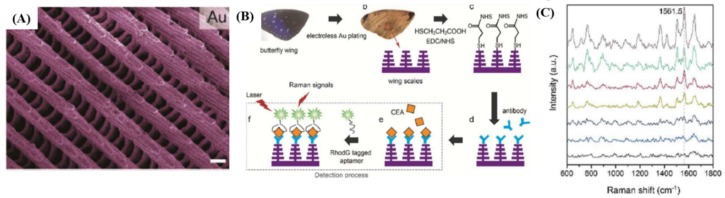
(**A**) Surface textures of the origin of butterfly wings covered with an Au layer of 40–70 nm thickness; (**B**) Schematic illustration representing the functionalization steps involved in the fabrication of butterfly wings for carcinoembryonic antigen (CEA) detection; (**C**) Surface enhanced Raman spectroscopy (SERS)-based CEA detection for five different clinical samples. (Reproduced with permission from Reference [36] published by the Royal Society of Chemistry).

In the same period, Porter et al. [35] have developed a multiplex SERS-based immunoassay platform for the detection of two pancreatic cancer biomarkers, here serum carbohydrate antigen 19-9 (CA 19-9)—this one being the only validated biomarker for pancreatic cancer—and matrix metalloproteinase 7 (MMP-7). The LODs of each target using SERS-based immunoassay were 2.28 pg∙mL^−1^ and 34.5 pg∙mL^−1^ for MMP-7 and CA 19-9, respectively, from spiked serum. Comparing these values to those obtained using the conventional ELISA technique, the increase in sensitivity was achieved, pointing out the possibility of using SERS in real-world samples. Recently, a CEA tumor biomarker was detected directly in human body fluids using a stable and highly reproducible Au butterfly wings platform as efficient SERS substrate [36]. With their natural 3D hierarchical sub-micrometer structures presented in Figure 2A, which is quite impossible to manufacture by conventional methods (e.g., photolithography, etc.), Au butterfly wings generate an effectively 3D enhanced SERS clinical detection of CEA. Specifically, the biosensing protocol proposed for the detection of CEA antigen is schematic illustrated in Figure 2B. To demonstrate SERS-based CEA detection ability of the functionalized Au butterfly wings platform (see Figure 2C), five different blood clinical samples were collected from patients at the Zhejiang Cancer Hospital, China. The authors have obtained acceptable accuracy for quantitative clinical CEA detection as compared to Abbott CEA reagent kits, demonstrating as such the feasibility of this platform to be further employed for the specific detection of multiple biomarkers.

Fluorescence spectroscopy is another well-known spectroscopic method playing a major role in selective detection of protein biomarkers. Similarly to the effect of increasing the Raman scattering of molecules, the presence of noble metal nanostructures can enhance the fluorescence signal from locally situated fluorophores. The phenomenon, known as metal-enhanced fluorescence (MEF), has gained considerable research interest in recent years [37]. An LSPR-based POC system was implemented by Zhou et al. [38] to enhance the excitation of the fluorescence labels for medical diagnostics. The detection configuration based on MEF is able to significantly enhance the sensitivity, up to 10-fold compared to a Au film for PSA biomarker detection. In particular, in the proposed POC system the fluorescence detection was recorded by a mobile phone camera.

Up to now, β2 microglobulin (β2M)—one of the very important biomarkers for cancers inflammatory disorders and kidney diseases [39]—was successfully detected by Preechaburana et al. [40] using a first angle-resolved surface plasmon resonance (SPR) detection system consisting of a single lab-on-chip device, an optical coupler (made up of polydimethylsiloxane (PDMS) rubber and epoxy) and a smartphone. Concretely, the illumination light source was provided in this case by the phone’s screen, while the front camera of the phone was used for reflected light collection. This as-developed system was able to detect pathophysiological range of β2M with an LOD of about 0.1 μg∙mL^−1^, a performance suitable for detection in the clinical range. Later, Liedberg et al. [41] have constructed a novel smartphone sensing platform that employ directly a built-in light emitting diode (LED) flash from the phone as light source and a camera as the detector. Then, the peptide-functionalized AuNPs with a diameter of 36 nm have been used to detect cTnI by real-time monitoring of the LSPR red-shift. The LOD of the smartphone-based sensing system, a fast, sensitive, and portable LSPR sensor, is estimated to be 50 ng∙mL^−1^, comparable to the value achieved with common SPR technique.

### 2.2. Solution-Based Homogeneous Immunoassays 

Homogeneous immunoassays, have attracted considerable attention recently, becoming useful platforms for the detection of biomarkers with prospects for easy automation and increase of analytical throughput [42]. Compared to typical heterogeneous immunoassays, which involve antibody immobilization, multiple steps of incubation and washing cycles, homogeneous immunoassays avoid multiple reactions and washing procedures before signal amplification and reading. Moreover, with the expanding use of nanomaterials in the biomedical field, colloidal plasmonic nanoparticles can play a significant role in the development of innovative, low-cost, rapid and easy-to-use homogeneous immunosensors in solution, due to their appealing plasmon-induced optical properties.

#### 2.2.1. Immunoassays Based on the Intrinsic Optical Properties of AuNPs

One important class of AuNPs-based immunoassays exploits the intrinsic optical response of AuNPs, such as absorption, scattering and intrinsic photoluminescence, to traduce the antigen-antibody binding events which can be further correlated with the concentration of biomarker in the analyzed sample. For example, the approach based on the spectral shift of LSPR band of AuNPs has been implemented as a transduction strategy in label-free immunoassays. In the early 2000s, Thanh and Rosenzweig [43] showed for the first time that AuNPs can be used to quantify antibodies in aqueous and serum samples based on an aggregation-based unique, sensitive, and highly specific immunoassay. Specifically, by monitoring the absorption change at 620 nm against anti-protein A concentration in serum samples, an LOD of 1 μg∙mL^−1^ anti-protein A was achieved. The results showed that the sensitivity of their AuNPs-based assay for the protein associated with the bacteria *Staphylococcus aureus* was comparable with the sensitivity of ELISA. Later, Wang and co-workers [44] brought a significant contribution to the field of immunosensors with real applicability in early diagnosis by designing a novel plasmonic biosensor based on Au nanorods (AuNRs) to detect the hepatitis B surface antigen (HBsAg), which indicates active viral replication of hepatitis B virus. By monitoring the wavelength shift of the LSPR peak of AuNRs induced by the immunological reaction, the biosensor showed a dose-dependence response ranging from 0.01 IU∙mL^−1^ to 1 IU∙mL^−1^, with an LOD of 0.01 IU∙mL^−1^. 

LSPR of AuNPs has been also exploited in the development of highly sensitive label-free fiber-optic biosensors for the detection of cancer biomarkers. For example, Li et al. proposed a new optical microfiber biosensor employing AuNPs as amplification labels, for the selective and sensitive detection of alpha-fetoprotein (AFP) from serum samples. After optimization, the detection scheme based on the LSPR shift occurring after an antigen-antibody binding event yielded an LOD for AFP of 0.2 ng∙mL^−1^ in PBS and 2 ng∙mL^−1^ in bovine serum, comparable to conventional assays [45].

AuNPs and LSPR-based biosensing approach has been recently explored as novel tool for the early diagnosis of prostate disease. Jazayeri et al. [46] established a novel approach for improving the efficacy and sensitivity of PSA by employing 25 nm colloidal AuNPs conjugated with anti-PSA antibody LSPR and monitoring the LSPR changes occurring as a consequence of AuNPs’ aggregation induced by antibody-antigen reaction. The same LSPR-based approach was exploited by Salahvarzi and coworkers [47] to develop an immunoassay based on AuNPs for detecting thyroid stimulating hormone (TSH), used for monitoring thyroid associated diseases, in human blood serum. The capture of TSH by anti-TSH monoclonal immobilized on the AuNPs surfaces by electrostatic adsorption induced a LSPR peak shift which was used as basis for determination of TSH antigen. A dynamic range between 0.4–12.5 mIU∙L^−1^ and a sensitivity of 1.71 L mIU^−1^ was obtained. Furthermore, TSH at a concentration of 6.2 mIU∙L^−1^ TSH is detected in human serum sample.

**Figure 3:**
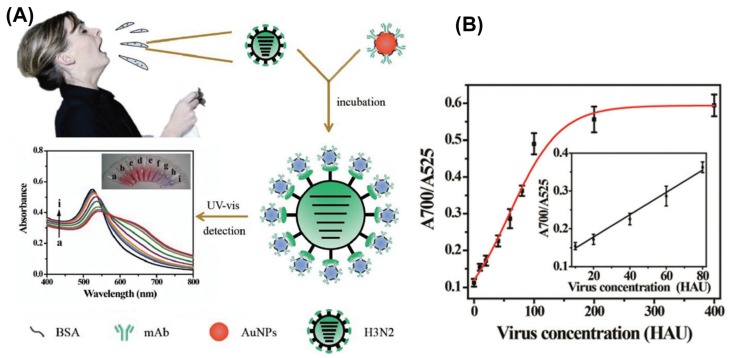
(**A**) Schematic illustration of the single-step colorimetric detection assay applied for the early detection of H3N2 influenza A virus (IAV) using Au nanoparticles (AuNPs); An obvious color change from red to purple and alteration of extinction spectrum is observed with the increase of H3N2 concentration; (**B**) Variance of absorption ratio of A700/A525 as a function of H3N2 concentration. (Reproduced with permission from Reference [53] published by the Royal Society of Chemistry).

The pioneering work of Mirkin [48] on the use of AuNPs as signal reporter for the selective detection of biological samples, based on the distance-dependent optical properties of AuNPs has boosted the expansion of AuNPs-based colorimetric assays. The optical properties of AuNPs are strongly dependent on the interparticle separation distance while their aggregation induces a significant shift in the extinction spectrum manifested as a color change of suspensions from red to purple. Most AuNPs-based colorimetric immunosensors are designed in such a way that binding of an analyte causes aggregation, and consequently a colorimetric response which is correlated with the concentration of the analyte in the analyzed sample. Such immunoassays have witnessed a rapid development due to several advantages when compared to traditional immunoassays, such as simplicity, rapidness, enhanced stability, reduction in nonspecific aggregation and no need for expensive or challenging instruments. This approach was used by Chen et al. [49] to develop a novel, simple and rapid label-free colorimetric assay based on fibrinogen and AuNPs for the highly selective and sensitive detection of thrombin, a biomarker of pulmonary metastasis, in blood plasma. The LOD obtained for thrombin—0.04 pM—is lower than those obtainable using other nanomaterial- and aptamer-based detection methods. In a similar way, neurogenin3 (ngn3), a marker for pancreatic endocrine precursor cells and associated with the development of diabetes, was quantitatively detected for the first time by Yuan and coworkers [50] using a label-free colorimetric immunosensor based on glutathione AuNPs. The electrostatic binding of positively charged ngn3 to the negatively charged anti-ngn3 labeled AuNPs induces the aggregation of NPs in the presence of salt resulting in a visible change of color and occurrence of a new optical band. The assay showed a linear response range of 50–300 ng∙mL^−1^ for ngn3 and a promising LOD of 20 ng∙mL^−1^. Recently, Liu et al. [51] developed a wash-free homogenous colorimetric immunoassay relying on the control of AuNPs growth for mediating the interparticle spacing in the protein-AuNPs oligomers. When applied for the detection of CEA, a commonly used clinical biomarker associated with various types of cancer, the assay displayed a linear dependence for 0–100 ng∙mL^−1^ range and an LOD of 5.66 ng∙mL^−1^ which is encouraging since in most individuals with cancer, CEA is often in the range from several to hundreds of ng∙mL^−1^. The group of Yang [52] showed that colorimetric immunosensing can be successfully applied for the detection of Amyloid β (Aβ), a key predictor of Alzheimer’s disease (AD). Specifically, AuNPs coated with N- or C-terminal antibody captured simultaneously Aβ inducing aggregation of NPs and a change of solution color from red to blue. The authors report good linearity within a range from 7.5 nM to 350 nM with an LOD of 2.3 nM, which is even better than other detection limits reported for Aβ. Colorimetric immunoassays can also be applied for the early diagnosis of viruses which is imperative for preventing their further spread and facilitate therapy. For example, influenza A virus (IAV) was detected using a colorimetric sensor based on AuNPs modified with monoclonal anti-hemagglutinin antibody (mAb), as schematically depicted in Figure 3A [53]. The single-step approach provides results by means of plasmon shift derived from the assembled mAb–AuNPs on the surface of subtype H3N2 virus occurring together with the change of color from red to purple, which can be quantified by absorption spectral measurements. The immunosensor revealed high specificity, accuracy comparable to clinically available HA inhibition tests, good stability and an LOD of 7.8 hemagglutination units (HAU) (see Figure 3B) [53].

The coherent radiation scattered by AuNPs when irradiated by light has also been exploited in diagnosis applications in conjunction with techniques like dynamic light scattering (DLS), resonance light scattering correlation spectroscopy (RLSCS) or resonance Rayleigh scattering (RRS). Liu et al. [42] were the first to develop a one-step homogeneous and quantitative immunoassay based on probing the dynamics of AuNPs by DLS. Spherical and nanorod-shaped AuNPs labeled with two different primary anti-PSA antibodies were exposed to PSA antigen leading to the formation of oligomers through sandwich type antibody-antigen-antibody linkages. A correlation was made between the relative ratio of dimers/oligomers versus individual AuNPs and the amount of antigen in solution. The assay was sensitive for PSA concentrations from 0.1 to 10 ng∙mL^−1^. In a similar way, Driskell et al. [54] developed a simple, rapid and sensitive method for quantitative detection of influenza A virus, using DLS and AuNPs as labels. DLS is used to measure the mean hydrodynamic diameter of aggregates induced upon the interaction of target virus with influenza-specific antibodies conjugated to individual AuNPs. This assay provides an LOD improved with up to 2 orders of magnitude compared to commercial diagnostic kits.

LikeDLS, RLSCS spectroscopy can be used to monitor AuNPs via the strong photon bursting phenomenon of single AuNPs occurring due to plasmon resonance scattering and Brownian motion of single NPs, however, in this case, in a small detection volume. Lan et al. [55] applied this approach to develop a fast and sensitive homogeneous sandwich immunoassay for PSA. Photon burst counting method can detect changes in the photon burst counts of AuNPs before and after immune reactions, when the formation of oligomers reduces the number of AuNPs. The relationship between the photon burst counts of AuNPs and PSA concentration was used to quantify the level of PSA in human serum samples. The authors report a linear behavior of the assay for PSA in the range of 1–1000 pmol∙L^−1^ and an LOD of 0.8 pmol∙L^−1^, which is good agreement with conventional ELISA assays. RRS takes place when the wavelength of Rayleigh scattering is located close to the absorption band of the scattering sample. The unique RRS of AuNPs was exploited for the first time by Cat et al. [56] in quantitative immunosensing applications. The assay was developed for the detection of transferrin, a clinical biomarker for protein-calorie malnutrition and a potential marker for diabetes. The detection based on the specific immune recognition between anti-transferrin antibody conjugated spherical AuNPs and transferrin from human serum samples allowed an LOD of 85 pM and a linear detection range from 85 pM to 3.4 nM.

**Figure 4:**
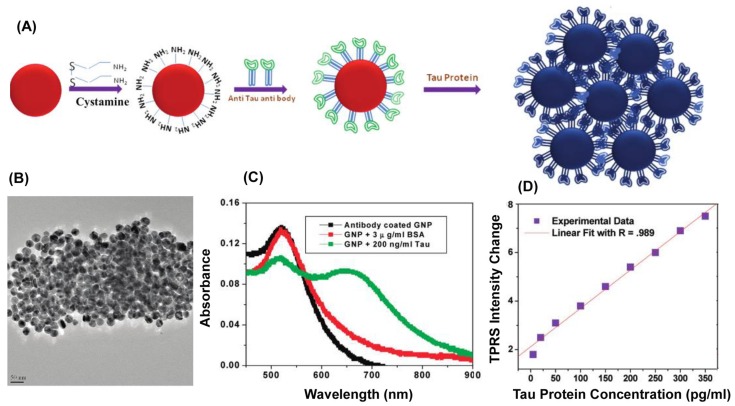
(**A**) Schematic representation of the synthesis of monoclonal anti-tau antibody-conjugated AuNPs and interaction with tau protein; (**B**) TEM image of anti-tau antibody-conjugated AuNPs after the addition of 20 ng∙mL^−1^ tau protein; (**C**) Extinction spectrum of monoclonal anti-tau antibody conjugated AuNPs in the presence of bovine serum albumin (BSA) and tau protein (200 ng∙mL^−1^); (**D**) Plot illustrating the linear correlation between two-photon Rayleigh scattering (TPRS) intensity and concentration of tau protein over the range of 5–350 ng∙mL^−1^. (Reprinted with permission from Reference [58] Copyright (2009) American Chemical Society).

Another type of scattering effect exploited in AuNPs-based detection assays is the Hyper-Rayleigh scattering (HRS), a nonlinear incoherent second-order light scattering. The HRS method relies on the fluctuations of the density or orientation of NPs, which break the centrosymmetry of isotropic media and create conditions of net frequency doubling. It has been shown that for antigen detection in an aqueous solution, the sensitivity by HRS intensity is 10 times higher than that by UV-Vis extinction spectroscopy [57]. Recently, the first label-free, fast, and highly sensitive immunoassay for the selective detection of AD’s biomarker based on HRS of AuNPs was reported [58]. In the presence of tau protein, anti tau labelled—AuNPs bind to each other, as depicted in Figure 4A, thereby producing aggregates visible in TEM (Figure 4B) and also indicated by the occurrence of a new absorption band at 670 nm (see Figure 4C). The authors show that the two-photon Rayleigh scattering (TPRS) from anti-tau antibody-coated spherical AuNPs increases linearly with the concentration of tau protein over the range 5–350 ng∙mL^−1^ (see Figure 4D) and that the assay can be successfully applied for detecting Alzheimer’s tau protein in the 1 pg∙mL^−1^ level which is about 2 orders of magnitude lower than cutoff values of 195 pg∙mL^−1^ for tau protein in cerebrospinal fluid [58].

Along with their abilities to scatter efficiently light, AuNPs have demonstrated over the years the capability to generate a strong intrinsic photoluminescence (PL), correlated with their well-defined plasmon resonances, hence enabling their probing by fluorescence spectroscopy toward sensing applications. Chen et al. [59] were the first to report the possibility of developing ultrasensitive immunoassays for the detection of proteins in blood serum based on probing the PL of AuNPs by fluorescence correlation spectroscopy (FCS). The idea was later embraced by Xu et al. [60] who developed a homogeneous immunoassay for the detection of thrombin from real samples based on a sandwich approach. When differently aptamer-labeled AuNPs were mixed with solutions containing thrombin, the affinity reaction caused the AuNPs to form dimmers or oligomers, which led to an increase in the diffusion time which was finally detected by FCS and correlated with thrombin concentration. Under optimal conditions, the assay provides an LOD of 0.5 nM.

#### 2.2.2. Immunoassays Based on AuNPs as Signal Quenchers/Enhancers

Due to the intense electromagnetic field generated at their surface and in close proximity when interacting with light, plasmonic NPs and of particular interest AuNPs, have the ability to modify (quench or enhance) the signal of molecules located in direct contact with them or nearby. For example, quenching of fluorescence by AuNPs have taken a considerable advance in the establishment of so-called fluoroimmunoassays. Such immunoassays exploit the ability of AuNPs to quench fluorescence more efficiently than organic quenchers not only via Förster resonance energy transfer but also by manipulating the radiative rate of nearby fluorophores [61]. In one of the first reports on fluoroimmunoassays employed for antigen detection, Ao et al. [62] combined AuNPs with magnetic NPs to develop a sensitive and specific system for the detection of α-fetoprotein (AFP), a serum marker of hepatocellular carcinoma. AFP was captured in between AuNPs and magnetic NPs both coated with anti-α-fetoprotein. The sandwich-type immunocomplex was then separated by a magnetic field while the supernatant containing unbound AuNPs, was used to quench the fluorescence of fluorescein isothiocyanate which was proportional to the concentration of AFP. The measurement range of the assay was from 15 to 400 ng∙mL^−1^ and the LOD was 12 ng∙mL^−1^, which is comparable to the normal concentration of AFP in serum (<20 ng∙mL^−1^). Later, the lowest LOD for cardiac thrombin T (cTnT) using a homogeneous sandwich assay based on AuNPs was reported. The principle of operation is based on the simultaneous interaction of cTnT with two different antibodies, one attached to AuNPs and the other labeled with fluorescent dyes, which induces the quenching of dye fluorescence, correlated with the concentration of protein. The LOD achieved was 0.02 nM (0.7 ng∙mL^−1^) [63]. Imposed by the urgent need to develop rapid, sensitive, and cost efficient tests for striking diseases, Guirgis and coworkers [64] have developed a rapid AuNPs-based fluorescence immunoassay suitable for malaria diagnosis from clinical samples. Fluorescence measurements show that the homogeneous assay can detect a concentration of Plasmodium falciparum protein 70 (PfHsp70) in infected human blood as low as 2.4 μg∙mL^−1^. The assay displayed a linear response within a range of antigen concentration from 8.2 to 23.8 μg∙mL^−1^. The same authors have later extended their work by combining fluorescence spectroscopy with a range of other techniques in order to obtain a detailed picture of the competitive immunoassay for malaria antigen detection in serum samples, based on fluorescence-quenching by AuNPs, especially under competitive binding conditions. More importantly, antigen-binding constants to AuNPs-antibody conjugates were determined for the first time [65].

In a different scenario, Chang and coworkers [66] exploited the localized electromagnetic field near AuNPs in combination with fluorescence to develop a fiber-optic biosensor based on localized surface plasmon coupled fluorescence able to detect AFP biomarker in human serum. Specifically, the proposed detection platform, integrating a sandwich immunoassay, is able to detect AFP concentration in PBS solution from 0.1 ng∙mL^−1^ to 100 ng∙mL^−1^ whereas a linear response between the fluorescence signals and the concentrations of AFP from 2.33 ng∙mL^−1^ to 143.74 ng∙mL^−1^ is observed in human serum.

Raman spectroscopy can be a very sensitive technique for quantitative detection and analysis of molecules, including disease biomarkers, at low concentration, even in solution. The enhanced electromagnetic field arising at the surface of plasmonic NPs, when the incident laser light is tuned to the plasmon resonance wavelength enhances the unique Raman “fingerprint” of molecules located in contact with metal or in close proximity, through SERS effect [67]. One of the first significant SERS-based immunoassays developed for diagnosis purposes exploits the combined properties of hollow gold nanospheres (HGNs) and magnetic beads for the detection of lung cancer marker CEA. HGNs and magnetic beads previously conjugated with polyclonal and monoclonal anti-CEA antibodies, respectively, bind in sandwich immunocomplexes in the presence of CEA antigen. Quantitative analysis was performed on samples obtained after removal of nonspecific binding HGNs using a bar magnet. The authors demonstrated that the developed SERS-immunoassay was quick and reproducible, providing an LOD of 1–10 pg∙mL^−1^, a value improved over 100–1000 times compared to ELISA [68]. In the following years, similar approaches based on magnetic capture and isolation of immunocomplexes for further signals processing have been reported. For instance, Chon and co-workers [69] demonstrated the simultaneous detection of two routine cancer biomarkers (i.e., CEA and AFP) by SERS under a single excitation wavelength by exploiting the same HGNs—magnetic beads combination. Two different Raman-tags attached separately onto HGNs enabled the detection of both biomarkers after the formation of immunocomplexes and magnetic-capture. The established trend to detect multiple disease biomarkers was also emphasized by Neng et al. [70] who established a sensitive SERS-based assay for the detection of multiple viral antigens. In contrast to similar studies, the strategy employed here consisted in performing SERS detection on Raman tag-labelled immunocomplexes magnetically concentrated after antigen capture. The proposed approach provided an LOD of 5 fg∙mL^−1^, which is 200–2000-fold greater then reported in previous studies on the detection of single antigens using magnetic capture assays. More recently, Lin and coworkers [71] have reported satisfactory results on the detection of CEA from real human serum through SERS, by employing easy-to-synthesize spherical AuNPs and magnetic core-shell AuNPs. Similar to previous studies, the selective detection of CEA antigen was achieved after the magnetic separation of immunocomplexes obtained from the interaction of antibody-functionalized Raman tag-labeled AuNPs and magnetic core shell AuNPs with CEA antigen from serum. The LOD was as low as 0.1 ng∙mL^−1^.

**Figure 5:**
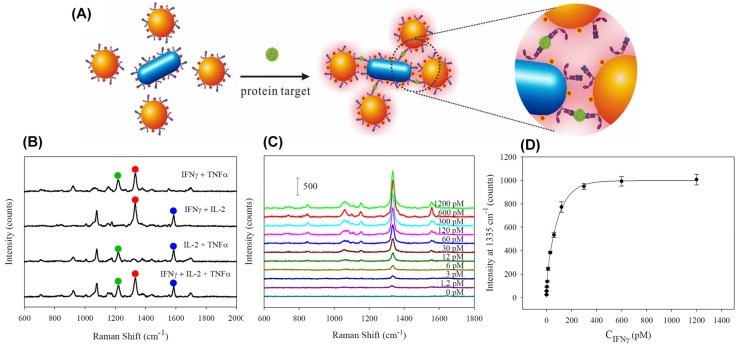
(**A**) Illustration of single-step SERS immunoassay based on plasmonic coupling enhancement via sandwich assembly; (**B**) SERS spectra obtained in response to different combinations of proteins targets; (**C**) SERS response for immunoassay of recombinant human interferon gamma (INFγ) protein with varying concentration; (**D**) The peak intensity at 1335 cm^−1^ as a function of INFγ concentration. (Reprinted with permission from Reference [73] Copyright (2013) published by American Chemical Society).

Simultaneously, others were able to develop SERS-based immunoassays that provided convincing results without the need to perform magnetic capture and isolation of immunocomplexes from the solution. For example, Neng et al. [72] claimed the first demonstration of a SERS-based immunoassay for the diagnosis of the infection with West Nile Virus using a single type of AuNPs. The strategy consisted in incubating AuNPs conjugated with antigen and blocking agent with unprocessed rabbit serum samples containing the immunoglobulin target analyte and bi-functional Raman tag/antibody binding reporter. The assay provided a minimum detection sensitivity of 50 pg∙mL^−1^ for targeted antibody in serum, a value significantly improved compared to ELISA. The SERS approach was also exploited by Wang et al. [73] to fabricate an innovative type of multiplexed immunoassay platform for the simultaneous detection of three cytokines, key mediators of various diseases. The strategy, called target-controlled assembly-based SERS immunoassay, consisted of using spherical and rod-shaped AuNPs labeled with monoclonal antibodies and Raman tags to create hot-spots for plasmonic enhancement via controlled sandwiched antigen-antibody assembly (see Figure 5A) [73].

By using the designed platform, the multiplexed quantification of the three cytokines (recombinant human interferon gamma (INFγ), interleukin-2 (IL-2) and tumor necrosis factor alpha (TNFα)) was possible, as shown in Figure 5B. By fitting the intensity at 1335 cm^−1^ versus concentration (see Figure 5C,D), an LOD of 0.5 pM was achieved for INFγ protein.

## 3. Metallic Nanoparticles in Composites with Bioactive Glasses and Glass Ceramics

### 3.1. Bioactive Glasses and Glass-Ceramics

Bioactive glass and glass ceramics are attractive materials for biomedical applications. One of the important advantages of these materials is the bone bonding ability [74], namely these materials are able to bind with living tissue by forming an apatite-like layer on the glass surface, following initial glass dissolution [75]. Leveraging these advantages, the bioactive glass particles and granules are used by orthopaedic surgeons and by dentists for orthopaedic bone grafting and dental bone regeneration. The Bioglass^®^ particles are also used in toothpastes for treating tooth hypersensitivity and bleaching treatments of teeth [76]. Another important application of bioactive glasses is for coatings of metallic implants such as hip prostheses and periodontal implants. The metals alone are bioinert, which means they are encapsulated with fibrous tissue after implantation [76].

In the last decade, different compositions of bioactive glasses were developed such as silicates [77,78,79,80,81,82,83], phosphates [84,85,86,87], borates [88,89], borosilicates [89,90,91,92], borophosphates [93,94,95], opening the possible applicability of these materials. For example, the borate glasses have faster bioactive kinetics than silicate materials [95,96] and the phosphate glasses are resorbable materials due to high solubility in aqueous media [94,97].

The first Bioglass^®^ was prepared by melt quenching method, by mixing various oxides, i.e., SiO_2_, P_2_O_5_, CaO, Na_2_O, melting under the crystallization temperature followed by rapid cooling [98]. With this simple preparation method, fully dense materials can be obtained. This limits their application in medicine [99], but they are very useful when the specific application does not require highly porous materials. With the appearance of the sol-gel method, this disadvantage of bioactive glasses was eliminated [100]. The reduced sintering temperature permits us to obtain porous materials and new glass compositions. The process is based on inorganic polymerisation reaction of metal alkoxides and metal salt precursors and it takes place in the following steps: hydrolysis and condensation reaction of molecules (sol formation), gelation (sol-gel transformation), aging and drying [100,101,102].

One of the applicability requirements of the biomaterials is to obtain a tissue engineered bone scaffold. The concept of a bone scaffold is that it can act as a three-dimensional temporary template to guide bone repair, thus the ideal scaffold can simulate the natural mechanism of human bone formation. Therefore, the bone scaffold must meet certain criteria such as: support of cell attachment, proliferation and differentiation, excellent bioactivity, good biodegradability, adequate microstructure, relevant structural-mechanical properties, irregular shape fabrication and commercialization potential [76,103,104]. Beside an important disadvantage, namely the low mechanical strength and decreased fracture resistance, bioactive glass and glass ceramic scaffolds have many of these properties. A promising approach for scaffold production is composite materials comprising a biodegradable polymeric phase and a bioactive inorganic phase, such as bioactive glass and glass ceramic [105]. Whereas the polymeric phase degradation shows the ideal rate of tissue degradation and keeps the space conducive for tissue in growth and vascularisation, the bioactive phase should promote bone growth [105]. 

Although the application of first bioactive glasses and glass-ceramics was in a skeletal system, nowadays these materials are also proposed for a wide range of application such as neuromuscular repair, artificial cornea, orbital implants, epithelial and cardiac tissue engineering, treatment of gastric ulcers and non-osseous cancer therapy [106]. Thus, beside the existing requirements of ideal scaffolds, other customized requirements appeared. For this reason, there is the desire to use the properties of nanoparticles (NPs) in scaffold engineering.

### 3.2. Role of Metallic Nanoparticles in Organism

It is known that the composition of bioactive glasses and glass ceramics determines their biological performance. Composite materials built from biomaterials and noble metal NPs are desirable for infection treatment and prevention. These types of structures combine the properties of each component resulting in a bioactive antibacterial and pro-inflammatory compound ideal for healing processes. Thus, incorporating specific ions or NPs into the glass composition can lead to further release of these species in the biological environment, modifying the material’s performance [78]. Metallic elements such as silver, copper and gold have been used as doping bioactive glasses referring to the specific biological effect of these metallic ions [107].

In both the melt quenching and sol-gel method the noble metal amount is conventionally indicated in the oxide form such as Ag_2_O, Au_2_O or CuO. To promote the thermal reduction of these metal ions leading to NPs formation, the glass powders are subjected to thermal treatments.

However, many studies have shown the biological response of bioactive glass composites with metallic NPs, as summarized in Table 1.

The different NPs incorporated into glass and glass-ceramics composition can have a major impact on the glass structure, bioactivity and biocompatibility. The resulted specific properties are applicable in tissue engineering and will be discussed in the next section.

#### 3.2.1. Silver Nanoparticles in Bioactive Glass and Glass Ceramics

Silver nanoparticles (AgNPs) have been employed in a broad range of applications due to their unique physical and chemical properties as it has been previously claimed that these particles could be used in various medical applications for reinforcement to tissue repair, for anti-inflammatory agents, for biosensors and hygiene [124,125,126,127,128].

The bioactivity properties of AgNPs are described by the antibacterial activity and healing enhancement effect of nano-silver. The bactericidal property of AgNPs has been debated for a long time and it is still unknown which is the main antimicrobial mechanism. Studies have proposed several mechanisms. The mechanism of antibacterial activity depends on the size of the NPs. When the NPs are smaller than 10 nm, they can penetrate through the cell wall, in this way damaging bacteria. When the NPs are bigger than 10 nm, the antibacterial activity can be explained by adhesion of the NPs to the bacteria surface resulting in cell wall damage [129,130]. Thus, the role of Ag content in the glass matrix is to reduce the microbial contamination. This property is also a requirement for the materials introduced into the living organism considering the increasing amounts of multi resistance bacteria stains, which are very often involved in hospital acquired infections.

The antibacterial effect of bioactive glasses doped with AgNP was demonstrated in many studies [112,113,114]. Magyari et al. [112] tested the antibacterial activity of borophosphate glasses with silver oxide (Ag_2_O) content using *Listeria monocytogenes*. For these materials, a good antibacterial effect appears when the glass contains minimum 0.2 mol% Ag_2_O (AgNPs with 10 nm diameter), but once Ag_n_ clusters appear (1.5 mol% Ag_2_O) this effect was diminished. Goh et al. [113] found that sol-gel silicate bioactive glasses with Ag_2_O content above 5 mol% prevented the *Escherichia coli* bacterial colonization effectively after 24 h. Fan et al. [111] used mesoporous bioactive glass as a template to deposit AgNP and found better antibacterial performance against *Staphylococcus aureus* and *Escherichia coli* when the AgNPs were distributed uniformly onto the surface.

**Figure 6:**
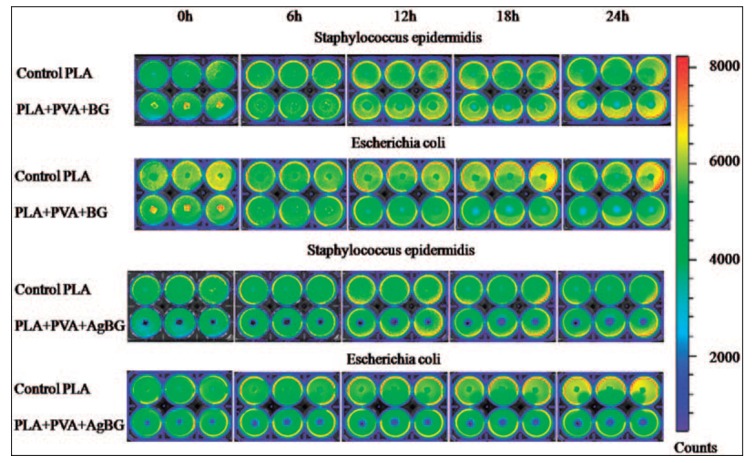
Bioluminescent signals from *Escherichia coli* and *Staphylococcus epidermidis* in response to the presence of polymer/bioactive glass and polymer/bioactive glass with Ag_2_O composites. (Reproduced with permission from Reference [114] published by Sage).

Vulpoi et al. [114] tested antibacterial activity of the composite of polymer-bioactive glass with Ag_2_O on genetically modified light-emitting bacteria *Escherichia coli* and *Staphylococcus epidermidis* by measuring the bioluminescence of the indicator strains (Figure 6). It was discovered that the polymer-bioactive glass composite with Ag content showed bactericidal effect, while inhibition of bacterial growth was also proved for the Ag-free polymer-bioactive glass composite.

The glasses and glass composites with AgNP can be prepared both with sol-gel and melt quenching methods by adding the Ag component [131] or can be introduced as AgNP in the structure of the composite [111,132,133]. The conventional melt quenching method applies silver nitrate (AgNO_3_) or silver oxide (Ag_2_O) as the precursor. This technique includes the mixing of the corresponding materials of the desired composition, melting in a temperature higher than 1200 °C and quenching the glass melt. In these glasses, the size and the dispersion of AgNPs was dependent on the used precursors, the concentration of Ag_2_O and the glass composition. Ahmed et al. [134] and Magyari et al. [125] used AgNO_3_ as starting materials for preparing phosphate glasses doped with 0.5–2 mol% Ag_2_O and 0.5–1 mol% Ag_2_O, respectively. It was demonstrated that, when the Ag_2_O content in the phosphate glasses was <0.5 mol% only Ag^+^ ions were present in the glass, while metallic Ag^0^ was obtained when Ag_2_O content exceeded 0.8 mol%. Baia et al. [135] produced phosphate glasses doped with 0.05–0.25 mol% Ag_2_O using and demonstrated the existence of AgNPs in the range between 1.5 and 5 nm. When the silver oxide content was between 0.7 and 3 mol% isolated spherical, 9 nm sized AgNPs were obtained in borosilicate glasses [136].

The sol-gel synthesis pathway is an alternative method for the preparation of bioactive glasses and can be described as a formation of an oxide network via polycondensation reactions of molecular precursors in a liquid [137]. Vulpoi et al. [109,110] and Goh et al. [113] prepared silicate bioactive glasses with Ag_2_O content between 2 and 8 mol% and 1–10 mol%, respectively and observed that when the Ag_2_O concentration was at least 2 mol% AgNPs were present and when the Ag_2_O content was higher (8–10 mol%) the obtained AgNPs possessed different sizes and shapes.

**Figure 7:**
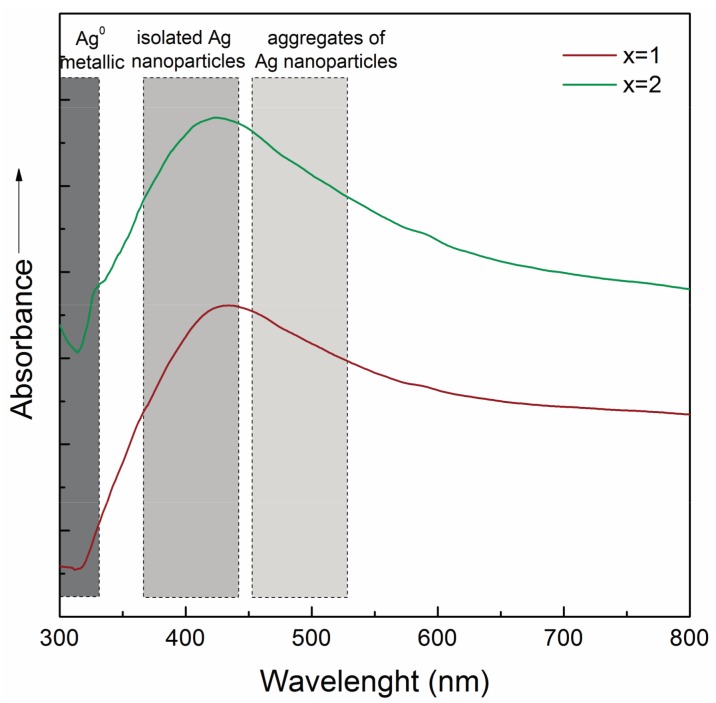
UV-vis spectra of the 60SiO_2_(32−x)CaO·8P_2_O_5_∙xAg_2_O samples.

The incorporation form of Ag can be demonstrated by Transmission Electron Microscope (TEM), UV-Vis absorption and X-ray Photoelectron spectroscopy (XPS). UV-Vis spectroscopy is the fastest and cheapest, with the disadvantage that it cannot determine the distribution, the exact size and shape of NPs. Since Ag atoms, ions, NPs and clusters exhibit different optical properties, thus these can be evidenced by means of UV-Vis spectroscopy. The electronic transition involving Ag^+^ ions is present in the 190–230 nm spectral range, while the electronic transition of Ag^0^ between 250 and 330 nm, and 400–500 nm, due to Ag SPR [138,139,140]. An example is shown in Figure 7 when the UV-Vis spectra of silicate bioactive glasses with Ag_2_O content are presented. It can be seen that the maximum is around 420 nm, which can be associated with the existence of very small, spherical Ag particles, formed inside the glass matrix.

The presence of AgNP on the surface can be also evidenced by XPS spectra and it is possible to determine the elemental composition of the outermost layer (10 nm depth). The size of AgNP can be estimated using the Ag 3d core level shifts, which were shown to be sensitive to the particle size [136,141].

The healing potential of AgNPs was mentioned by Wong et al. [127] where AgNPs were proposed to facilitate proliferation and migration of *keratinocytes*, to reduce formation of collagen by *fibroblasts* and to modulate the number of cytokines produced.

However, pure AgNP may be toxic to any living organism; therefore, the material’s surface functionalization is an important issue. Also, without any steric stabilization, AgNPs may aggregate easily because of their high surface energy. Some of the best coatings agents used to achieve biocompatible nanocomposites that also act as stabilizing agents are polymers.

Formation of AgNPs involves two major steps: reduction of the Ag precursor and growth of the AgNPs. The chemical reduction of Ag precursors in homogenous liquid media with assistance of capping molecules is an excellent approach to obtain stable Ag colloids in a quick and inexpensive manner [142]. The most popular reducing agents are applied in aqueous media; however, for a more complex synthesis procedure organic media can be used. The capping molecules are applied because they strongly bond to the surface of AgNPs to prevent the NPs from aggregation. Popular materials that can be used as both reducing and stabilizing agents are polymers. 

#### 3.2.2. Gold Nanoparticles in Bioactive Glasses and Glass Ceramics

Incorporation of AuNPs in bioactive glass matrix can widen the medical applicability of these materials: diagnostics, therapy, prevention and hygienic applications [116,119,143]. This depends on the promising properties of the AuNPs, such as biocompatibility, facile surface modification, stability and optical properties, which can be tuned by particle size and shape, surface chemistry and charge [116,143]. Concerning the toxicity of AuNPs it was found that the colloidal particles (3–100 nm) do not have any toxic effect on cell cultures, while the 1–2 nm sized AuNPs are considered dangerous due to the possibility of irreversible binding to biopolymers in the cells [143]. At the same time, one must consider the upper size limit of penetration via the hematoencephalic barrier which is between 5 and 20 nm.

Many studies demonstrated that bioactive glasses with Au content do not have toxic effect [116,117,119] and their bioactivity is not affected by the presence of AuNPs [116,117,118]. One promising application of the bioactive glass with AuNP is to serve as drug delivery agents [116], as it was also shown by Jayalekshmi et al. [119]. The bioactive glass–polymer composite was incorporated in the voids formed by the removal of bone tumors for controlled drug release to suppress the further tumor formation, and to enhance bone growth.

The effect of AuNPs content in glass systems on cell cultures depends on the used cells. Aina et al. [116] tested Hench’s bioactive glass with AuNPs on *Human osteoblast* cells and it was observed that the samples did not show any negative effects. However, when the glasses contained Au*^n^*^+^ species an increase of lactate dehydrogenase leakage and malondialdehyde production was observed.

The good proliferation rate of *Human keratinocytes* cells were obtained on bioactive glasses with AuNPs, which were very close to the value obtained in the presence of free AuNPs [117]. These results show the preservation of AuNPs’ properties in the glass system and open an application in wound healing processes.

The AuNPs size and maximum concentration in the glass matrix is dependent on the preparation method of the glass and on the starting materials. In the case of melted glasses the upper limit concentration of AuNPs is lower compared to that from the glass obtained by sol-gel method, due to the poor solubility of Au [144]. Aina et al. [116] added Au into the Hench’s Bioglass 45S5, prepared by melt quenching method using gold (III) chloride trihydrate (HAuCl_3_∙3H_2_O) as starting material. They obtained the glass samples with metallic Au isolated atoms and with small AuNPs (5 nm).

**Figure 8:**
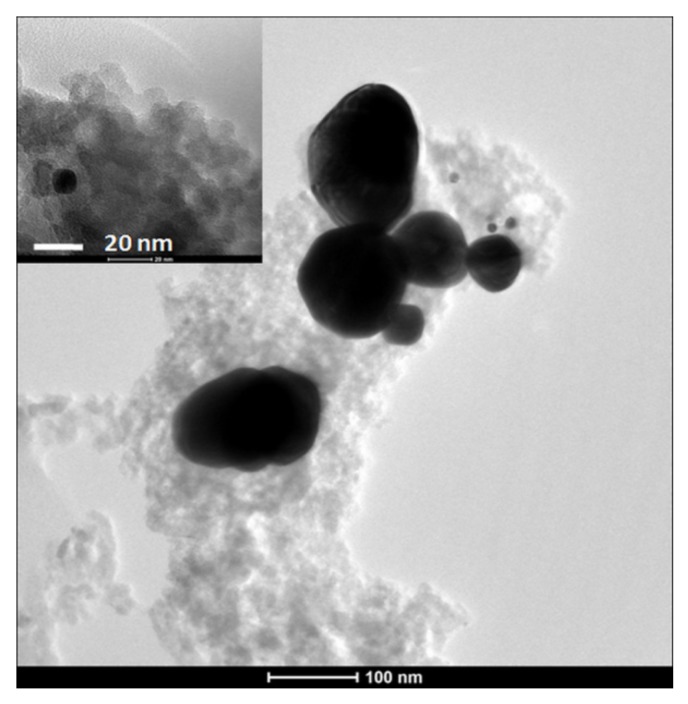
TEM micrographs of sample 60SiO_2_∙31.85CaO∙8P_2_O_5_∙0.15Au_2_O (mol%). (Reproduced with permission from Reference [117] published by Elsevier).

In the sol-gel derived glasses, the Au can be introduced only during the sol-gel preparation in two ways. One method is to use the HAuCl_3_∙3H_2_O as a precursor and the AuNPs in the glass matrix are obtained by thermal treatment [115,118]. However, this way the size of the NPs was reported to be greater than 25 nm, and Au*^n^*^+^ (*n* = 1 or 3) species together with Au^0^ in the form of isolated atoms were also present [115,118]. In another method the previously prepared AuNPs were introduced into the samples. Jayalekshmi et al. [119] reported that the AuNPs were incorporated in the sol-gel derived bioactive glass-polymer composite by functionalization via the amine linkages. In another study, the Pluronic stabilized spherical AuNPs were embedded in bioactive network during the sol-gel preparation process [117]. In the obtained glass matrix, a part of AuNPs keeps its original size, but a higher amount of NPs with size of about 100 nm also formed as a result of heat treatment (Figure 8).

#### 3.2.3. Copper Nanoparticles in Bioactive Glasses and Glass Ceramics

Like AgNPs, copper nanoparticles (CuNPs) also demonstrated size-dependent antibacterial activity with low toxicity and good stability [145], which can be used to enhance a biomaterial’s qualities. It is known that the Cu ions have angiogenesis response, thus can promote the bone growth and mineralization [146]. By introducing Cu in the glass structure, one can obtain materials with angiogenesis response and with antibacterial activity. The presence of Cu metal as Cu*^n^*^+^ species on surface of the glass can be also used to bind different biomolecules in order to obtain drug delivery systems [121].

Several studies revealed that CuNPs containing silicate glasses did not show any bioactivity alteration [120,121,122]. The antibacterial activity of silicate glass with Cu has been demonstrated by Popescu et al. [122] by obtaining samples with good antibacterial effects against *Staphylococcus aureus*. Esteban-Tejeda et al. [123] used CuNPs containing soda-lime glasses against *Escherichia coli* and *Micrococcus luteus* and obtained good antibacterial effect also. Aina et al. [121] proved the presence of CuNPs also on the glass surface, suggesting that this material is a good candidate for immobilization of organic molecules through the covalent bonding with their SH groups.

The introduction of CuNPs into the melt derived glass matrix is more difficult, due to the significant susceptibility of Cu to oxidation, relative to other noble metals (Ag, Au). Several methods have been used to eliminate this disadvantage, such as embedding monodispersed CuNPs into sepiolitefibres [123], incorporation of a reducing agent (tin) together with CuO in the matrix [147] and ionic exchanged processes [148].

In the sol-gel derived glasses for the Cu content the copper(II) nitrate trihydrate (Cu(NO_3_)_2_·3H_2_O) is usually used as a precursor and the CuNPs in the glass matrix is obtained by thermal treatment [120,121,122,149]. Using different thermal treatment conditions (temperature and atmosphere) it is possible to obtained glasses with Cu in different oxidation states. Bonici et al. [120] produced silicate glasses with Cu and after different thermal treatment obtained e.g., Cu^2+^ containing glasses at 600 °C, CuNPs of spherical shape into glass matrix at 700 °C (controlled atmosphere) and a mixed Cu^2+^/Cu^+^/Cu^0^ NPs-containing glasses at 1050 °C.

## 4. Photocatalytic Application of Gold Nanoparticles

A variety of the noble metals used for catalytic purposes have been widely and intensively studied. Therefore, a specification is needed to organize the available information. Au is from a catalytic point of view the “elder” element although, considering that in specific areas of catalysis it was introduced in the last three decades. Photocatalysis is one of the previously mentioned areas, where Au can play multiple roles, mostly as a co-catalyst together with semiconductors. This area shows a continuous increase in interest, which is why the present section focuses on the last three years of Au mediated photocatalytic reactions. It should be mentioned that other metals and noble metals are also used as co-catalysts in photocatalytic reactions. The present section will focus on gold, to have a compatibility with the section discussing Au SPR-based detection applications.

**Figure 9:**
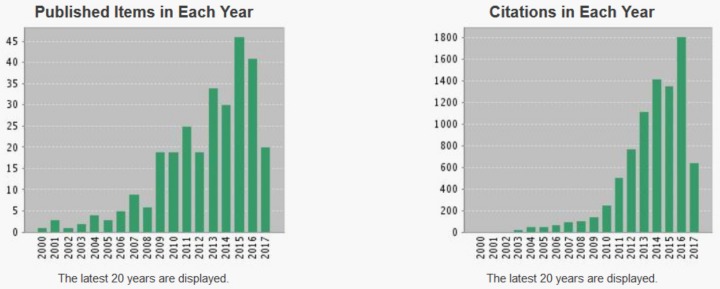
The increasing trend of Au applicability in photocatalysis (Web of Knowledge, https://apps.webofknowledge.com, access date: 22 May 2017).

As the official scientific database, the Web of Science shows a high number of publications appeared which contained the words “photocatalysis” and “gold”, and the trend is still increasing, due the vast number of available photocatalytic materials (Figure 9). Furthermore, the application of binary, ternary and quaternary composite photocatalysts also increases dramatically.

**Figure 10:**
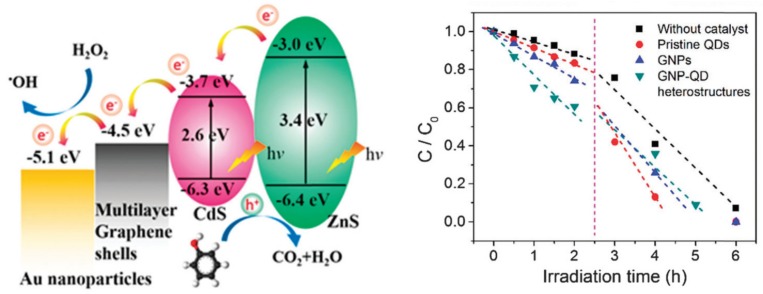
The charge transfer mechanism (**left**) of the quaternary composite formed from ZnS/CdS/graphene shells and Au; the degradation of phenol (**right**) in the presence of the quaternary composite (Reproduced with permission from Reference [150] published by the Royal Society of Chemistry).

One of the most intensively studied materials was graphene. This material does not necessarily show photocatalytic activity by itself, but it can act as a 2D conductor material of the photogenerated electrons. This construction was applied when ZnS/CdS/graphene shells and Au were combined. The graphene acted as the electron transfer element (Figure 10), while Au was the terminal component, which transferred the electron to the liquid phase. This is possible as Au does not show any overpotential in the electron transfer processes towards electrophile species. This mechanism contributed to the H_2_O_2_ assisted degradation of phenol under UV-A light [150].

Graphene (G) and graphene oxide (GO) nanostructures were included in composites which were active in visible light [151], making the prepared material as a potential indoor active (when no UV light is accessible) photocatalyst. GO played the same role as G in the previous case [150], while in this case methylene blue was the model pollutant, and the photocatalyst was Si supported SiO_2_. The additional role of Au was the plasmonic enhancement, which provided visible light absorption.

As it was shown above, carbon containing nanostructures were applied as electron transfer agents between the photoactive semiconductor and Au. Based on the facts listed above it is an obvious issue to exploit the properties of Au directly by using it together with other noble metal NPs or by creating Au containing alloys.

**Figure 11:**
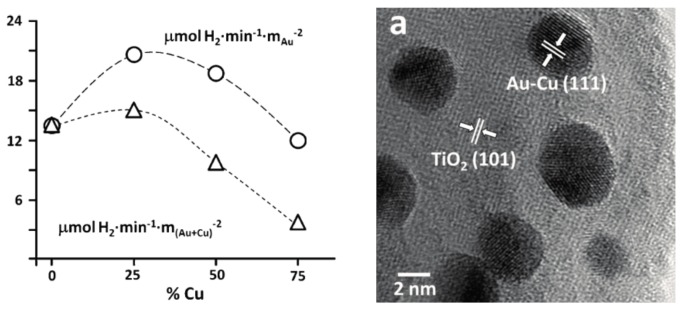
The enhanced activity of Cu/Au—TiO_2_ NPs in photocatalytic hydrogen production (**left**); the high-resolution transmission electron microscopy (HR-TEM) micrograph of the used composite material (**right**). (Reproduced with permission from Reference [152] published by Springer).

The main reason for alloying can be multiple, including efficiency increase and cost-related aspects as well. This was the case when Cu-Au alloys were obtained and deposited on the surface of TiO_2_. The presence of Cu was beneficial in both catalytic and economic aspects [152]. The first aspect can be attributed to the increased interparticle electron density transfer between Cu and Au and it was proven by photocatalytic hydrogen production experiments (Figure 11).

When two non-alloy NPs were loaded on the surface (Au and Pt) of a semiconductor, such as g-C_3_N_4_ an interesting synergistic effect was observed [153]. Usually, Pt deposited semiconductors show higher hydrogen production rates compared to Au due to well-known reasons [154]. However, the presence of both noble metal NPs induces a further enhancement of the activity surpassing even the performance of the Pt containing composite material [153]. It seems that bi-metallic alloyed NPs or two metal containing non-alloyed composite material shows the same trend concerning the activity enhancement, pointing out a necessary content optimization in both cases. Introducing a metal nanoparticle can confer other interesting properties to the semiconductor. Sometimes, besides the charge separation, also the physical separation of the catalysts from suspensions is required. For this purpose Ni NPs can be efficiently applied. This was demonstrated recently in Au and Ni containing ZnO nanorods [155].

Noble metals are usually referred to as metallic and sometimes amorphous NPs. However, in the case of Au rare cases can be found in the literature where the main photocatalyst is an oxide of a noble metal. The representative example is the case of PdO, which was used with AuNPs for the efficient removal of tetrodotoxin [156]. The electron mediator was graphene oxide as discussed in the first case [150].

Most of the scientific work carried out in the field of photocatalysis in the last three years focused on Au and semiconductor-based materials. One of the interesting non-oxide semiconductors applied was CdS a material with enhanced visible light activity. The experiments carried out with this material showed two approaches. One of them used Au as a core, CdS as the shell along with a third component (e.g., TiO_2_). The next approach was using bare CdS containing AuNPs on its surface.

The first approach used the Au and CdS in a Z-scheme photocatalyst construction, meaning that the core-located Au acted more likely as an internal charge separator. The first electron transfer step is from the main photocatalyst towards Au, which injects it in the valence band of CdS. This induces a high electron density on the surface of CdS on the high hole density on the main semiconductor. This mechanism can be applied in photocatalytic hydrogen reduction [157] or in the photoreduction of CO_2_ into CH_4_ or CO [158]. The Z-scheme approach is valid for many important photocatalytic materials, including TiO_2_ [158], WO_3_ [157], g-C_3_N_4_ [159], etc. The role of Au in this case is very similar to the role of GO and G mentioned earlier, providing electron transfer assistance [150]. If the classical approach was used, then CdS acts as the visible light active photocatalyst, while Au is the electron transfer end-point [160].

**Figure 12:**
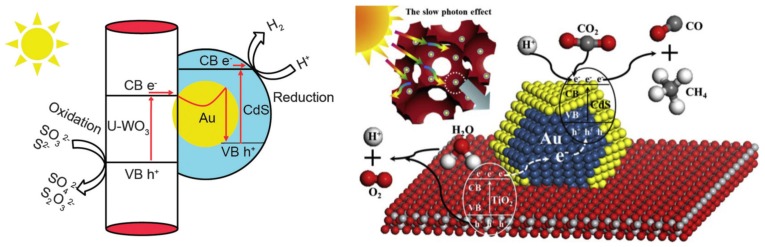
The application of Au in Z-scheme photocatalysts for photocatalytic hydrogen production (**left**) (Reproduced with permission from Reference [157] published by the Royal Society of Chemistry) and CO_2_ photoreduction (**right**) (Reproduced with permission from Reference [158] published by Elsevier).

**Figure 13:**
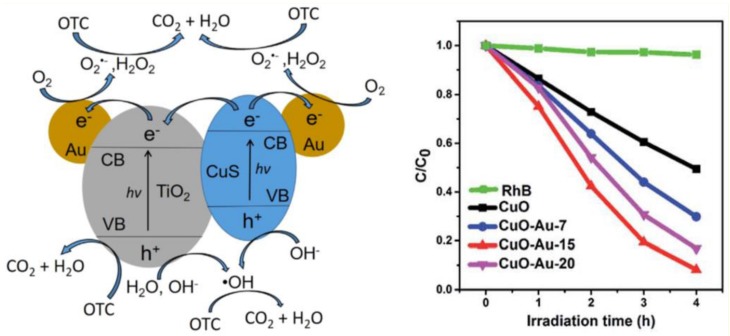
The role of CuS, TiO_2_ and Au in ternary composites in the degradation of oxytetracycline(OTC) (**left**) (Reproduced with permission from Reference [161] published by American Chemical Society) and the photocatalytic performance of CuO-Au composites in the photodegradation of rhodamine-B (**right**) (Reproduced with permission from Reference [162] published by American Chemical Society).

The synergistic effect discussed in the case of Cu/Au [152] and Au/Pt [153] was not a special case, as this could be valid when a co-catalyst semiconductor and Au are applied together with a photoactive component. A representative case could be the Au-CuS-TiO_2_ ternary composite, where Au played its usual end-point electron transfer role, while CuS was involved in the absorption/activation by visible light/charge separation, while TiO_2_ was the main photocatalytic component. This nanoarchitecture offered the possibility to work quite efficiently under simulated sunlight [161] for the degradation of oxytetracycline (OTC). For the role of each component, a mechanism was proposed (Figure 12). Besides CuS, CuO can be also applied together in Au, where the classical composite component contributions can be considered [162]. However, in this case, a detailed Au content optimization was also carried out and showed that in each case the content of the individual components was crucial, as showed by the degradation curves of rhodamine-B (Figure 13).

CuS can be exchanged for a UV active component, such as SnO_2_. The main essence of the charge separation mechanism remains the same, meaning that the overall UV efficiency was enhanced. This could be a logical step as visible light active photocatalysts show in most cases low quantum efficiency. The efficient degradation of methylene blue was demonstrated by this composite design route [163].

Most of the Au containing composites was used in aqueous media, although there are interesting applications for gas phase organic compound degradation. Propylene can be efficiently oxidized in the presence of a photocatalyst and Au to CO_2_ and water. For this, molecular oxygen is needed directly. However, it was shown that even if the oxygen receives the electron from Au, the adsorption of ethylene itself can be hindered by AuNPs with inappropriate size (>10 nm). In some cases this will lead to the saturation and even decrease of the number of exposed active sites on the base photocatalyst [164].

**Figure 14:**
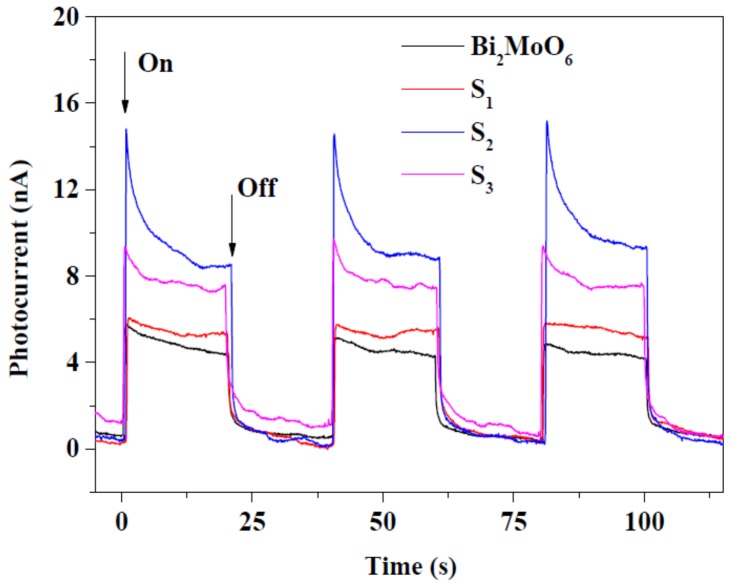
The photocurrent response of Bi_2_MoO_6_ and the composite materials (S1–S3) (Reproduced with permission from Reference [165] published by Elsevier).

Until now, simple compounds were considered as composite partners for Au in different photocatalytic processes. Modern photocatalysis is unimaginable without mixed oxides, a new category of photocatalytic materials, with special properties. Among them, Bi_2_MoO_6_ is one of the newly investigated ones. Together with GO, Bi_2_MoO_6_ and Au are showing an extraordinary visible light response, shown in the photocurrent values (Figure 14) and enhanced degradation results for the removal of rhodamine-B [165].

**Figure 15:**
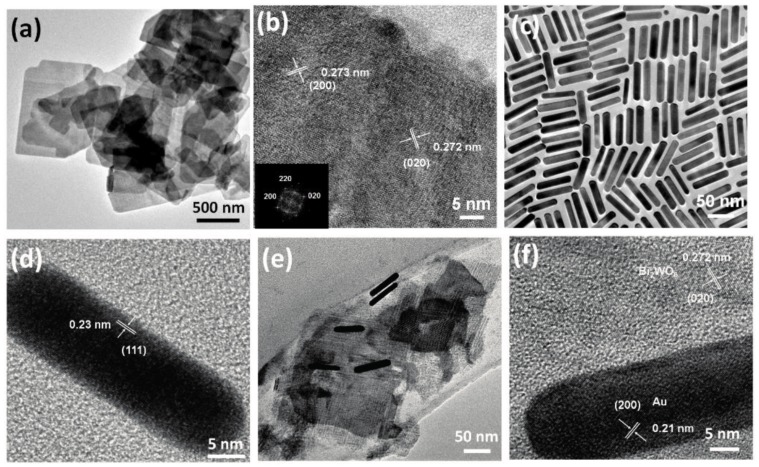
(**a**) TEM micrographs of Bi_2_WO_6_nanosheets; (**b**) HRTEM micrograph of Bi_2_WO_6_ nanosheets. Inset: Fourier transformed electron diffraction pattern of Bi_2_WO_6_; (**c**) TEM and (**d**) HRTEM micrographs of Au nanorods (AuNRs); (**e**) TEM and (**f**) HRTEM micrographs of Au NR/Bi_2_WO_6_ heterostructures. (Reproduced with permission from Reference [166] published by John Wiley and Sons).

The enhanced visible lights response is one of the key properties of Bi_2_O_3_∙M*_x_*O*_y_* mixed oxides. Bi_2_WO_6_ is also a member of the above-mentioned category, but in the case of this material also near-infrared (NIR) photocatalytic activity in the degradation of methyl orange was achieved, with the help of rod shaped AuNPs. The as obtained composites contained Bi_2_WO_6_ nanoplates and Au as shown in Figure 15. The NIR activation was attributed to the plasmon band position of the Au nanorods which were located in the NIR (band maximum at 900 nm) [166]. This is rather important as the sunlight contains a large amount of IR/NIR light as well.

**Figure 16:**
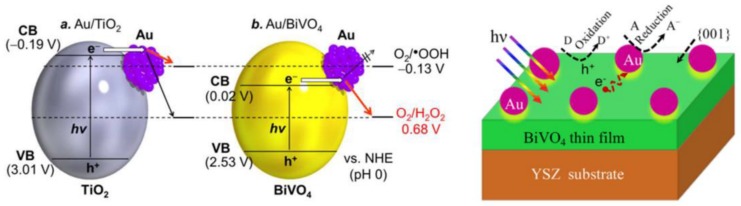
The mechanism of H_2_O_2_ generation (**left**) (Reprinted with permission from Reference [167] Copyright (2016) published by American Chemical Society) and the enhanced electron transfer mechanism, when shape-tailored BiVO_4_ was applied (**right**) (Reproduced with permission from Reference [168] published by Elsevier).

Not only can the photocatalytic enhancement be achieved by the deposition of Au, but in specific cases it can contribute as the mediator in the generation reactions of reactive compounds, such as H_2_O_2_ [167]. As the position of the valence band and the conduction band of BiVO_4_ permits the production of O_2_, which in the presence of H^+^ and an electron source (photoelectrons generated by the semiconductor and accumulated and transferred on the surface of Au) can be transformed to H_2_O_2_ (as shown in the equations below), and this can be converted to OH, an extremely reactive species. Furthermore, this charge separation mechanism can be further enhanced if the BiVO_4_ crystals’ morphology is controlled and manipulated to obtain exposed {001} facets which can separate in the first instance spatially the photogenerated electrons, facilitating the faster transfer towards the AuNPs [168] (Figure 16).

2H_2_O + 4h^+^ → O_2_ + 4H^+^ (1)

O_2_ + 2H^+^ + 2e^-^ → H_2_O_2_ (2)

Some of the mixed oxides can crystallize relatively easy in the form of hierarchical microstructures built from smaller nanocrystals. This structure is usually capable of generating a relatively high amount of charge carriers, which can recombine relatively easily. If Au was used, the activity of these nanostructures can be enhanced as it was proven for the case of Bi_2_O_2_CO_3_ [169]. Furthermore, if the aspect ratio of Au was manipulated, then the light absorption and photoactivity of Bi_2_O_2_CO_3_ can be further tuned [170]. The same strategy was proven to be valid also in the case of BiOX photocatalyst, which emphasizes the important role of Au in the enhancement of the photoactivity of hierarchical nanostructures [171,172].

**Figure 17:**
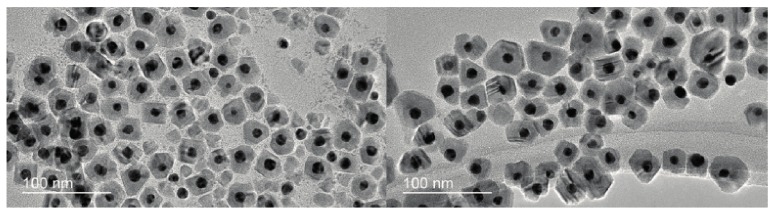
TEM micrographs of Au/Cu_2_FeSnS_4_ core/shell NPs (Reprinted with permission from Reference [173] Copyright (2015) published by the American Chemical Society).

As it was presented until now, only simple semiconductors and mixed oxides were considered as partners for Au in photocatalytic reactions. However, in recent years, non-conventional mineral-like photocatalysts were reported in the literature. Among them, an interesting example is that of Cu_2_FeSnS_4_ a quaternary chalcogenide, which was already applied in solar light conversion devices. Together with Au as a core, Cu_2_FeSnS_4_ can act as an extremely efficient water-splitting agent [173]. The construction plan of the composite was quite similar to the one applied in the case of CdS, when Au was applied in Z-scheme photocatalytic systems [157]. The build-up of the composites is shown in the TEM micrographs (Figure 17). Another interesting case was the Au/Bi_2_O_3_/FeVO_4_ ternary composite [174], where the Au showed the same role as in the case of BiVO_4_ [167].

**Figure 18:**
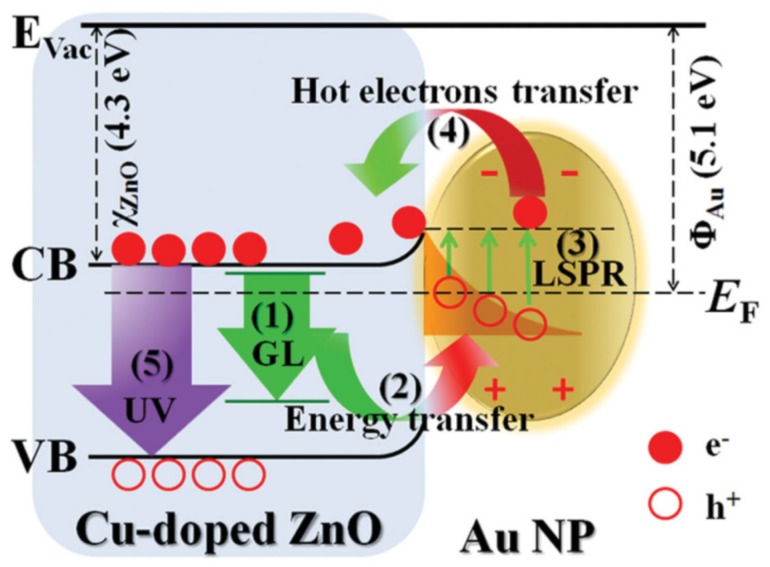
Schematic illustration of the possible mechanism of the UV enhancement involving the processes of (1) virtually generating green luminescence (GL) at Cu dopant, (2) transferring energy from the virtual GL of Cu-doped ZnO to localized surface plasmon resonance (LSPR) of Au NPs, (3) exciting LSPR of Au upon receiving the transferred energy from the virtual GL, and generating plasmonic hot electrons as a result of nonradiative decay of the LSPR, (4) transferring hot electrons from Au to the conduction band of Cu-doped ZnO, and (5) excitons radiative recombination at Cu-doped ZnO into UV emission (Reproduced with permission from Reference [176] published by John Wiley and Sons).

Until now, those materials were considered with Au, which did not cover most of publications. The second most dominant Au containing photocatalytic composite is the one with ZnO. This material presents a major advantage compared to the other semiconductors, namely that it can be easily shape-tailored using a vast number of synthesis methods. Therefore, the effect of Au can be different for each of the obtained crystal morphologies. One of the most frequent geometries of ZnO is the rod/wire form [175,176]. In this construction, several beneficial properties of the composites can be exploited. If un-doped, the Au/ZnO nanorod composite is an excellent visible light active photocatalyst [177] but when doped with Cu it shows an enhanced photoluminescence spectrum [176]. The mechanism of enhanced photoluminescence is presented below on Figure 18.

Another crystal geometry which is quite abundantly used in case of ZnO is sheet-/plate like. The ZnO structure in this case is also crystalline, while the deposited AuNPs were situated on the surface of sheet. The composite build-up shows similarities with graphene-based composite materials. The charge carriers can travel on the surface of the semiconductor to the Au nanoparticle, which is again the end-point electron transfer entity. As the recombination was inhibited, high degradation rates and efficient H_2_ production was observed [178]. Nevertheless, the presence of Au on a specific semiconductor surface can be achieved by selective deposition of specific crystallographic planes, similar to BiVO_4_ [168]. In this case [179], the electron rich crystallographic plane will also host the Au. In the case of ZnO this is the so-called polar facet.

Hierarchical ZnO materials are also possible, as in the case of Bi_2_WO_6_, permitting a high amount of charge carrier generation, while the presence of Au can efficiently hinder the recombination process. This approach can yield high efficiency photocatalysts with an activity of 10 times higher than commercial powders [180].

**Figure 19:**
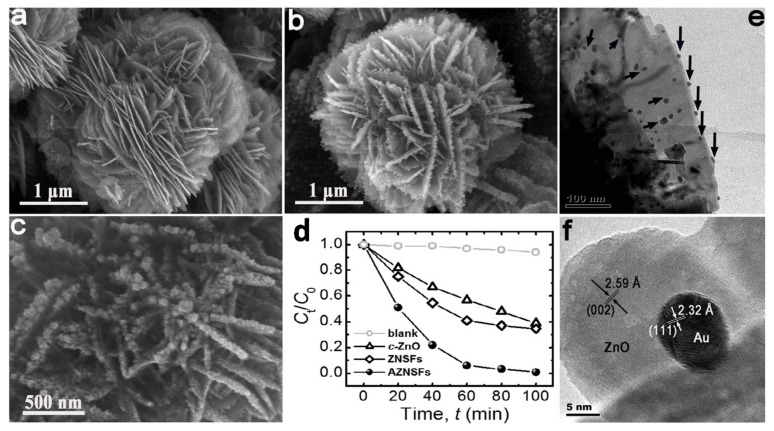
Hierarchical ZnO nanostructures (**a**,**b**) with AuNPs (**c**,**e**,**f**) used in the degradation of rhodamine-B (**d**) (Reproduced with permission from Reference [180] published by Elsevier).

Crystallographic plane development is also a focus for ZnO as stated previously. Also, 3D particles are a subject for this technique. The {002} plane family is also one of the most active ones in case of ZnO for photocatalytic applications, that is why obtaining particles with this specific plane was attempted (Figure 19). Obviously, the presence of Au can maximize the exploitability of these facets as it was shown by Ranasingha and coworkers [181]. In some cases, the approach listed previously for CdS and chalcogenides were proven to be useful when focusing on the synthesis of core-shell Au-ZnO NPs [182].

The most abundant semiconductor oxide which was used together with Au is TiO_2_. Even in the last three years, when a high variety of other semiconductors appeared, this oxide still dominates the scientific work in the field of photocatalysis and Au. This subject may constitute separately the essence of a separate review paper. 

**Figure 20:**
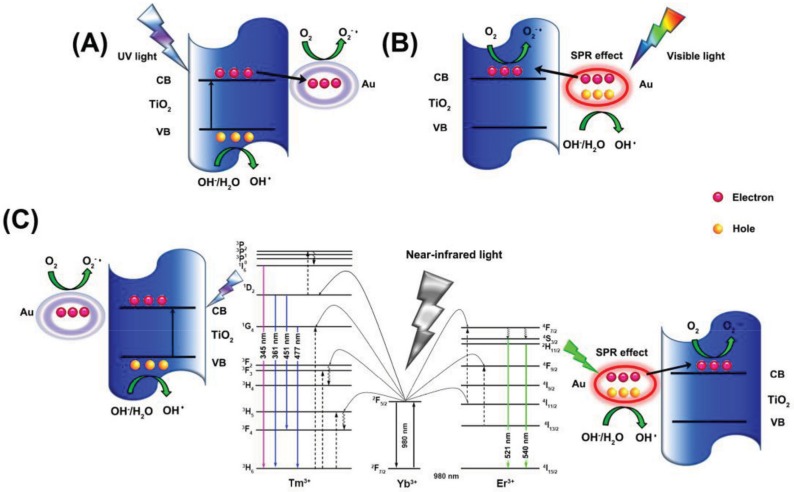
A schematic illustration of the photocatalysis mechanisms under (**A**) UV; (**B**) visible; and (**C**) NIR irradiation, respectively.(Reproduced with permission from Reference [185] published by John Wiley and Sons).

Also in the case of this material, the shape manipulation of AuNPs was essential [183,184], where the electron transfer properties of Au was the determining issue. However, TiO_2_ can sometimes be present along with a quite unconventional material, such as NaYF_4_ together with Au. This material is among the rarest which is active in the UV, visible and near infrared region [185]. Each of the irradiation regions activates the composite differently, as shown in Figure 20. When UV irradiation is applied, the active component is TiO_2_, while Au acts as the charge separator. When visible light was the irradiation source, the SPR effect generated hot electrons were injected in to the conduction band of titania, causing visible light activity. In the case of near-infrared irradiation a double excitation was considered, as NaYF_4_ provoked UV and visible light fluorescence. 

It was also considered that, besides the above-mentioned shape-tailoring approaches, the novelty concerning TiO_2_-Au nanocomposites will decrease with time. However, innovative approaches are still published, including the sandwiched TiO_2_-Au-TiO_2_ structures [186]. In this approach, the ternary structure was refused and just TiO_2_ was used. When sunlight was used, the already discussed SPR mechanism and the charge separation mechanism were considered simultaneously.

## 5. Concluding Remarks and Future Perspective

Sometimes, at first sight, the three research areas (nanosensing, bioceramics and photocatalysts) discussed in this work have very few aspects in common. However, there are bridging materials such as AuNPs, which through their LSPR and high electron transfer properties can be applied in various ways. This shows that a solution to a problem in one field can be solved from the knowledge acquired from the another. An interesting example can be that the NIR/IR activity of photocatalysts was achieved by using Au nanorods which were already applied in different immunosensors. As this example shows, it is mandatory to present the applicability of some nanomaterials (in our case dominantly Au) in multiple fields to point out the possibilities and solutions scattered throughout different research fields.

The development of highly sensitive, selective, and reliable Au-based nanosensors will undoubtedly further enable early diagnosis, improve diseases treatment, increase the overall survival and diminish societal costs. Moreover, considering the Au’ surface versatility which provides many possibilities for functionalization, the capability to finely tune their surface properties, size, shape as well as aggregation state together with the ability to improve their plasmonic response, we are confident that different design Au-based nanosensors will be more and more translated into clinical use in smart disease diagnostics.

Bioactive glass and glass ceramics represents an important group of biomaterials usable as implant, tissue engineering scaffold for hard and soft tissue regeneration or in drug delivery systems, but may be also designed to have at least double function: the one given by the bioactive glass or glass ceramic itself, namely to favor the tissue repair and at least one of the followings given by the incorporation of metal NPs (i) selective release in the body of NPs with antibacterial effect (Ag, Au, Cu); (ii) release in the body of functionalized NPs as carriers for drug delivery (Au functionalized with different biomolecules through Au-N weak linkage), (iii) protein immobilization (Au functionalized with different biomolecules strong Au-S bonds) and (iv) enhance proliferation rate of keratinocytes cells (Au).

It is clear that from the photocatalytic point of view noble metals can play multiple roles, including charge separation, surface plasmon resonance enhanced visible light activation and electron mediator. The most intensively investigated material for this purpose was Au. In the last three years, it was shown that classical approaches have been applied repeatedly (e.g., the combinations with TiO_2_), with different strategies (shape-tailoring of the AuNPs and that of semiconductors, core-shell and sandwich construction modes), while at the same time new materials (e.g., NaYF_4_) or high performance electron conductors (e.g., graphene) were applied as composite partners. It was shown that the most efficient route to enhance the photoactivity of Au containing composites is the application of ternary-, quaternary composites, in which each of the components has a specific role, starting from the charge generation, electron mediator and ending in the terminal electron transfer material, while in some cases adsorption enhancers were also included in the composites. The future of this research area looks “golden” as these approaches are taken/applied and developed by the ones working in this field, while simultaneously new materials are included and developed. The most problematic issue, however, remains the price of Au. In this aspect intensive research is also being carried out in order to replace Au partially or totally with other cheaper alternatives.

## Figures and Tables

**Table 1:** Effect of metallic nanoparticles (NPs) on organism: summary of literature studies.

NP	Composites	Biological Response In Vitro/In Vivo	References
AgNP	phosphate glasses	in vitro bioactivity	[108]
	silicate bioactive glass	in vitro bioactivity; protein adsorption	[109,110]
	mesoporous bioactive glass	antibacterial activity	[111]
	borophosphate glass	antibacterial activity	[112]
	bioactive glass	in vitro bioactivity; antibacterial activity	[113]
	bioactive glass-polymer	in vitro bioactivity; antibacterial activity	[114]
AuNP	bioactive glass	in vitro bioactivity; cytotoxic effect, cell viability	[115,116,117,118]
		cytotoxic effect, cell viability	[116,117]
	polymer-bioactive glass	biocompatibility, cell viability	[119]
CuNP	bioactive glass	in vitro bioactivity	[120]
	bioactive glass,	in vitro bioactivity	[121]
	bioactive glass-ceramics	in vitro bioactivity; biocompatibility; cell viability, antibacterial activity	[122]
	soda-lime glass	antibacterial activity	[123]
